# Screening and Validation of the Hypoxia-Related Signature of Evaluating Tumor Immune Microenvironment and Predicting Prognosis in Gastric Cancer

**DOI:** 10.3389/fimmu.2021.705511

**Published:** 2021-06-25

**Authors:** Jun-Peng Pei, Chun-Dong Zhang, Maimaititusun Yusupu, Cheng Zhang, Dong-Qiu Dai

**Affiliations:** ^1^ Department of Gastrointestinal Surgery, The Fourth Affiliated Hospital of China Medical University, Shenyang, China; ^2^ Department of Gastrointestinal Surgery, Graduate School of Medicine, The University of Tokyo, Tokyo, Japan; ^3^ Cancer Center, The Fourth Affiliated Hospital of China Medical University, Shenyang, China

**Keywords:** hypoxia, gastric cancer, nomogram, prognosis, tumor immune microenvironment

## Abstract

**Background:**

Hypoxia is one driving factor of gastric cancer. It causes a series of immunosuppressive processes and malignant cell responses, leading to a poor prognosis. It is clinically important to identify the molecular markers related to hypoxia.

**Methods:**

We screened the prognostic markers related to hypoxia in The Cancer Genome Atlas database, and a risk score model was developed based on these markers. The relationships between the risk score and tumor immune microenvironment were investigated. An independent validation cohort from Gene Expression Omnibus was applied to validate the results. A nomogram of risk score model and clinicopathological factor was developed to individually predict the prognosis.

**Results:**

We developed a hypoxia risk score model based on SERPINE1 and EFNA3. Quantified real-time PCR was further applied to verified gene expressions of SERPINE1 and EFNA3 in gastric cancer patients and cell lines. A high-risk score is associated with a poor prognosis through the immunosuppressive microenvironment and immune escape mechanisms, including infiltration of immunosuppressive cells, expression of immune checkpoint molecules, and enrichment of signal pathways related to cancer and immunosuppression. The nomogram basing on the hypoxia-related risk score model showed a good ability to predict prognosis and high clinical net benefits.

**Conclusions:**

The hypoxia risk score model revealed a close relationship between hypoxia and tumor immune microenvironment. The current study potentially provides new insights of how hypoxia affects the prognosis, and may provide a new therapeutic target for patients with gastric cancer.

## Introduction

Gastric cancer is a public health burden, ranking fifth in global incidence and fourth in mortality among all cancers (1). Therapeutic strategies are still based on the American Joint Committee on Cancer (AJCC) tumor/node/metastasis (TNM) staging system (2, 3). However, due to the high heterogeneity of gastric cancer, patients with similar clinicopathological characteristics could have different prognosis, suggesting the current TNM staging system are inadequate for predicting prognosis and risk stratification (4, 5). Therefore, it is clinically important to develop a novel biomarker to better guide clinical treatment and improve prognosis.

Tumor cells always grow faster than their blood vessels. Owing to the inadequate blood supply, the supply of oxygen and nutrients to the tumor cells is unbalanced, thereby forming a hypoxic microenvironment (6–9). Hypoxia is one of the characteristics of tumor microenvironment (TME) that can lead directly to the malignant characteristics, including tumor proliferation, migration and invasion, resulting in a poor prognosis (10–12). Previous studies have shown a significant relationship between hypoxia and poor prognosis of GC (13, 14), and hypoxia plays a key role in metastasis (15). In the hypoxic microenvironment, hypoxia-inducible factors (HIFs) are key transcription factors that allow cancer cells to survive under hypoxic conditions and promote tumor progression (16–18). Multiple genes transcribed by HIFs, including Glut1, KLF8, VEGFA, ITGβ1, etc., can promote GC metastasis and lead to poor prognosis (19).

TME is the internal environment in which tumor cells are produced and survive. It is composed of immune cells, endothelial cells, mesenchymal cells, inflammatory mediators and extracellular matrix molecules (20, 21). The immunological components of the TME can inhibit or promote tumor development (22). Recently, the significance of hypoxia in promoting tumor immunosuppression and immune escape has received increasing attentions (23, 24). It is important to understand the potential mechanisms that are involved between hypoxia and the tumor immune microenvironment. Therefore, the establishment of a hypoxia-based signature may help to identify the potential prognostic value of hypoxia, and improve the comprehension of the immunogenomic profile of gastric cancer.

Here, we established a hypoxia-related signature related to prognosis by The Cancer Genome Atlas (TCGA) data base, which was validated by the Gene Expression Omnibus (GEO) data base. Potential mechanisms of the hypoxia-related signature were further investigated.

## Materials and Methods

### Patients

The Clinical data (375 cancer and 72 non-cancerous samples) and FPKM RNA-seq data from TCGA data base (https://www.cancer.gov/tcga) was applied as a screening cohort. The data of 433 cancer samples (GSE84437) from GEO data base (https://www.ncbi.nlm.nih.gov/geo/query/acc.cgi) was applied as a validation cohort (25). RNA-seq and microarray data included were transformed [log_2_(x+1)] and normalized by the “sva” and “limma” packages of R software. The baseline characteristics of the screening and validation cohorts are shown (**Supplementary Table S2**).

### Development of a Risk Score Model

Univariate analysis was firstly applied to identify potentially hypoxia-related genes that have a statistically significant difference of prognosis in gastric cancer patients from TCGA data base. Least absolute shrinkage and selection operator (LASSO) method was then applied to shrink the scope of gene screening (26). Finally, Cox proportional hazards analysis was used to identify highly hypoxia-related genes. The risk score formula was constructed as: Risk score = (∑coefficient_x_ * expression of signature gene_x_) (gene_x_ indicated the identified genes). The regression coefficient was obtained from Cox proportional hazards analysis. The patients of gastric cancer were divided into a high-risk and a low-risk groups by the cut-off value of the median risk score.

### Tumor Immune Microenvironment

To investigate the relationships between risk score and TME, the ESTIMATE algorithm was applied to determine immune score, stromal score, ESTIMATE score, and tumor purity of individual patient in the screening and validation cohorts (27). Wilcoxon test was applied to compare the differences between the high-risk and low-risk groups in terms of immune score, stromal score, ESTIMATE score, and tumor purity. The TIMER web server (http://timer.cistrome.org/) was applied to analyze the correlations between signature genes and immune cells. The TIMER algorithm was used to assess the abundances of six immune infiltration cells (B cells, CD4^+^ T cells, CD8^+^ T cells, neutrophils, macrophages and dendritic cells) and tumor purity (28).

The ssGSEA method was used to the transcriptome to assess immune cell infiltrations (29). We obtained a set of marker genes including immune cell types, immune-related pathways and functions (30). We used the R package called “GSVA” to perform ssGSEA to obtain the normalized enrichment score (NES) of each immune-related item.

### Development and Assessment of a Predictive Nomogram

Univariate analysis and Cox proportional hazards analysis were conducted on risk score of target genes and patient clinicopathological characteristics to determine independent prognostic factors related to prognosis. The predictive nomograms were developed by including all independently prognostic factors.

### GC Cell Lines and Tissue Samples

The human gastric epithelial cell line GES-1 and gastric cancer cell lines AGS, SGC-7901, HGC-27, MKN-45 and MGC-803 were purchased from the Chinese Academy of Sciences (Shanghai, China). Cells were cultured in RPMI 1640 medium (HyClone, Logan, UT, USA) with 10% fetal bovine serum (FBS, Invitrogen) and 1% penicillin/streptomycin in a humidified atmosphere of 5% CO_2_ at 37°C. Totally, 39 pairs of gastric cancer together with their adjacent non-cancerous tissues (> 5 cm away from cancer tissue) were collected. This study was approved by the Ethics Committee of the Fourth Affiliated Hospital of China Medical University (EC-2021-KS-043). All patients included in this study provided written informed consent in accordance with the Declaration of Helsinki.

### Quantitative Real-Time PCR Analysis

Total RNA was extracted using Trizol reagent (Invitrogen, Eugene, OR) and was used to synthesize cDNA using Prime-Script RT Master Mix (TaKaRa, Shiga, Japan), and quantitative real-time PCR (qRT-PCR) was performed by TaKaRa SYBR^®^ Premix Ex Taq™ (TaKaRa, Shiga, Japan). All primers of qRT-PCR were listed in **Table S1**.

### Statistical Analyses

All analyses were performed by R version 4.0.2 (http://www.R-project.org). The calibration curve and area under the curve (AUC) were used to evaluate the predictive performance of the predictive nomogram. The clinical benefit was further evaluated by the decision curve analysis (DCA) (31, 32). An independent validation cohort was applied to validate these findings. All tests were two-sided, and a *P* value less than 0.05 was considered as statistically significant.

## Results

### Patient Characteristics

The baseline characteristics of the screening and validation cohorts are shown in **Table S2**. In the screening cohort, a total of 234 (63.1%) patients were male and 137 (36.9%) were female. Among them, 17 (4.6%) patients were T1, 74 (19.9%) were T2, 177 (47.7%) were T3, and 103 (27.8%) were T4 cases. 117 (31.5%) patients were N0, 97 (26.1%) were N1, 79 (21.3%) were N2, and 78 (21.1%) were N3 cases. Accordingly, 8 (2.2%) patients were Grade I, 126 (34.0%) were Grade II, and 237 (63.8%) were Grade III. Considering the TNM staging system, 46 (12.4%) patients were stage I, 119 (32.1%) were stage II, 165 (44.4%) were stage III, 41 (11.1%) were stage IV cases.

In the validation cohort, a total of 296 (68.4%) patients were male and 137 (31.6%) were female. Among them, 11 (2.5%) patients were T1, 38 (8.8%) were T2, 92 (21.2%) were T3, and 292 (67.5%) were T4 cases. Accordingly, 80 (18.5%) patients were N0, 188 (43.4%) were N1, 132 (30.5%) were N2, and 33 (7.6%) were N3 cases.

### Screening of Hypoxia-Related Risk Signature in Gastric Cancer

**Figure 1:**
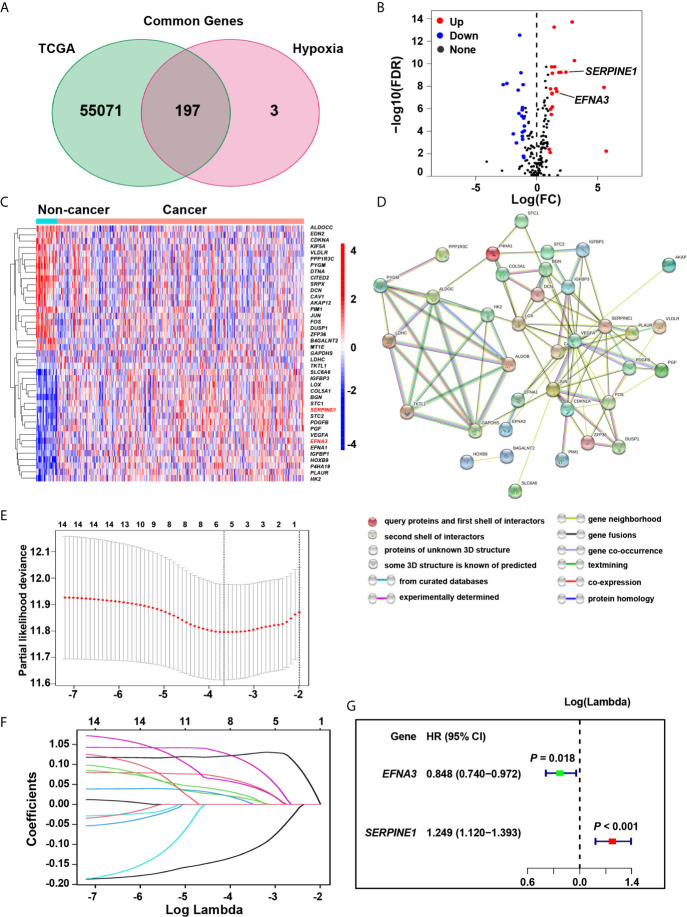
Identification of the hypoxia risk signature. **(A)** The Venn diagram shows the hypoxia-related genes in TCGA. **(B)** The Volcano plot for differentially expressed genes (DEGs) in cancer and non-cancer tissues. **(C)** The heatmap plot for DEGs in cancer and non-cancer tissues. **(D)** The PPI network visualizes the interaction between these DEGs. **(E, F)** The LASSO method identified five genes associated with prognosis. **(G)** The Cox proportional hazards analysis identified the hypoxia risk signature.

The hallmark hypoxia-related 200 genes, was obtained from the Molecular Signatures data base (MSigDB version 6.0). Among them, the TCGA data base contains 197 hypoxia-related genes, and 41 differentially expressed genes (DEGs) have been identified (**Table S3** and **Figures 1A–C**). To better visualize the interactions between these hypoxia genes, the STRING online data base was used to analyze the protein-protein interaction network (**Figure 1D**). We evaluated the hypoxia-related genes in the screening cohort, and identified 14 of the 41 genes that were significantly associated with prognosis (all *P* < 0.05, **Table S4**). The LASSO method was further used to analyze these 14 genes, which minimized the potential over-fitting problem and established the minimum standard. Five of the 14 genes in the model are under the optimal adjustment parameter (λ) (**Figures 1E, F**). Finally, the Cox proportional hazards analysis confirmed that two genes (*SERPINE1*, *EFNA3*) (**Figure 1G**) met the proportional hazard hypothesis and were finally used to establish the following risk score model: Risk score = (0.223 * expression level of *SERPINE1*) + (–0.165 * expression level of *EFNA3*). Of the two signature genes, *SERPINE1* was a risk DEG, and *EFNA3* was protective. The risk score of individual patient was calculated and all patients were classified into a high-risk and a low-risk groups based on the median risk score.

### Prognostic Ability of Hypoxia-Related Risk Score in Gastric Cancer

**Figure 2:**
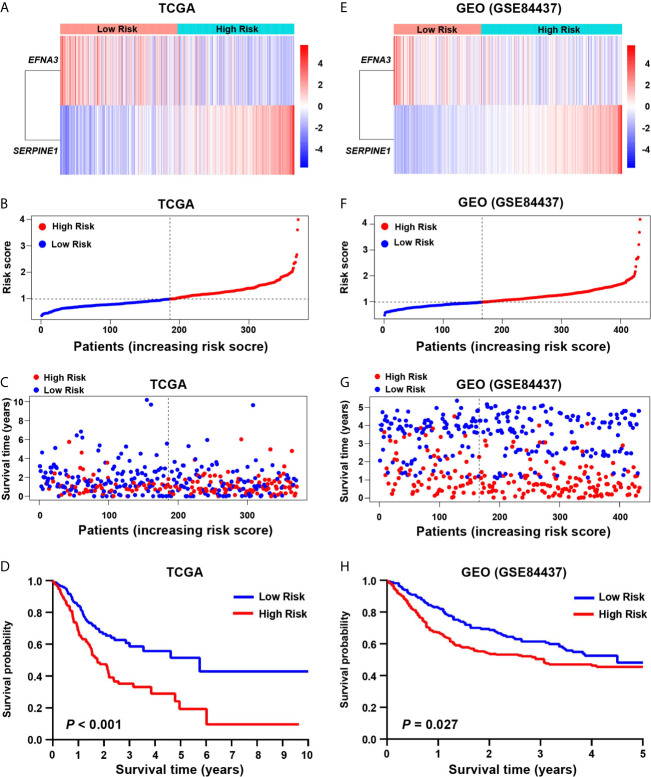
Prognostic value of the hypoxia risk signature in gastric cancer. **(A, E)** Heatmaps of the prognostic signature in the screening (TCGA) and validation (GEO) cohorts. **(B, F)** Patient risk score in the screening and validation cohorts. **(C, G)** The status distribution of patients in the high-risk and low-risk groups in the screening and validation cohort. **(D, H)** Kaplan-Meier analysis of patients in the high-risk and low-risk groups in the screening and validation cohorts.

Hypoxia usually promotes an aggressive tumor phenotype, so the prognostic ability of the hypoxia-related risk score was explored. In the high-risk group of the screening cohort, the heatmap showed that the expression of *SERPINE1* was up-regulated and *EFNA3* was down-regulated (**Figure 2A**). The mortality rate in the low-risk group was significantly lower than that in the high-risk group (**Figures 2B, C**). Kaplan–Meier analysis indicated that the prognosis of the low-risk group was significantly superior than that of the high-risk group (log-rank test, *P* < 0.001) (**Figure 2D**). Similar results were found in the validation cohort (**Figures 2E–H**).

### Hypoxia-Related Signaling Pathways

**Figure 3:**
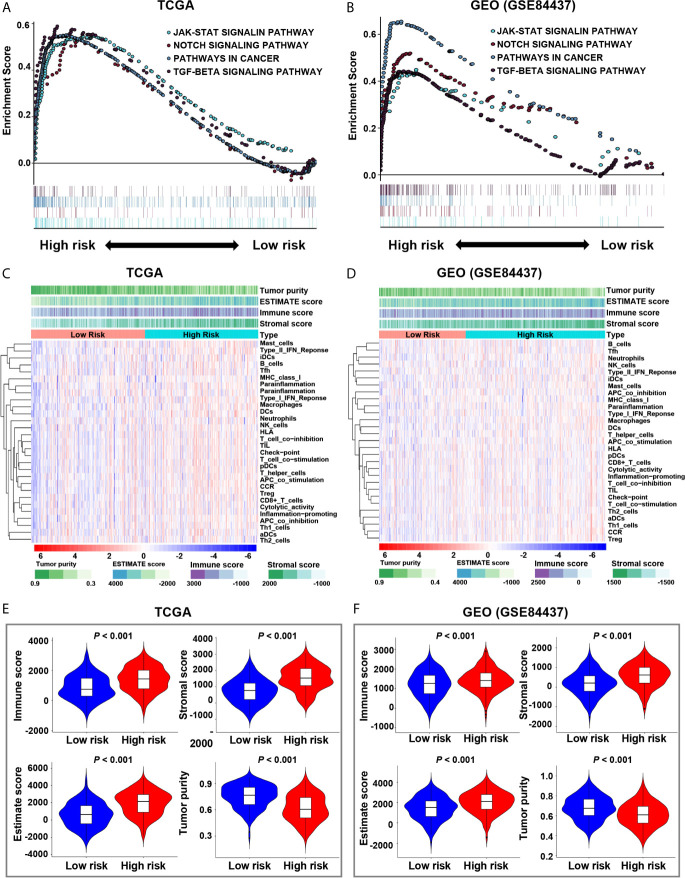
Enrichment of pathways related to hypoxia and analysis of tumor immune microenvironment. **(A, B)** The enrichment plots show the signaling pathways related to hypoxia in the screening and validation cohorts. **(C, D)** The heatmaps show 29 immune-related gene sets, immune score, stromal score, ESTIMATE score and tumor purity in the screening and validation cohorts. **(E, F)** The relationship between risk score and immune score, stromal score, ESTIMATE score, and tumor purity in the screening and validation cohorts.

In the screening cohort, we used GSEA to analyze the signaling pathways activated in the hypoxia-related high-risk group. In the high-risk group, the JAK-STAT signaling pathway, NOTCH signaling pathway, pathway in cancer, and TGF-β signaling pathway were activated (**Figure 3A**). These signaling pathways are related to the stimulation of tumor proliferation, migration, invasion, anti-apoptosis, Epithelial-Mesenchymal Transition (EMT), immune escape and drug resistance. These results have been confirmed in the independent validation cohort (**Figure 3B**).

### The Correlation Between Risk Score and TME

The ESTIMATE analysis showed that the immune score, stromal score and ESTIMATE score were significantly positively correlated with the risk score in both the screening and validation cohorts, while tumor purity was significantly negatively correlated with the risk score (**Figures S1A–H**). It also indicated that the immune score, stromal score and ESTIMATE score of the high-risk group were significantly higher than those of the low-risk group (*P* < 0.001), while the tumor purity of the high-risk group was significantly lower than that of the low-risk group (*P* < 0.001, **Figures 3C–F**).

### The Correlation Between Risk Score and Immune Cell Subtypes

**Figure 4:**
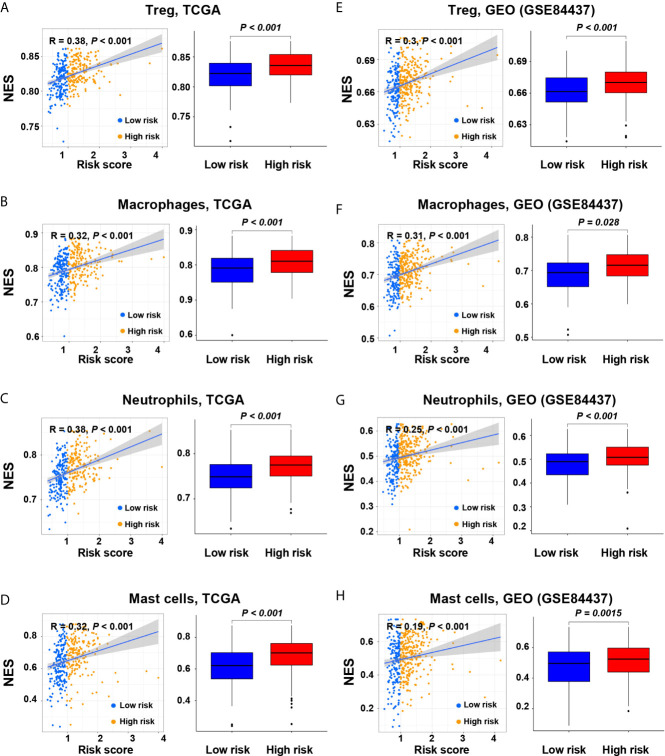
Correlation of the risk score with immune cell subtypes in the screening and validation cohorts. **(A, E)** Regulatory T cells; **(B, F)** Macrophages; **(C, G)** Neutrophils; **(D, H)** Mast cells.

As the tumors of the high-risk group were proved to be infiltrated with a large number of immune cells, we further analyzed the subtypes of infiltrating immune cells. It indicated that the levels of immune cell infiltration in the high-risk group, including regulatory T cells, macrophages, neutrophils, and mast cells, were higher than those in the low-risk group (*P* < 0.05, **Figures 4A–H**). Accordingly, the high-risk group reflects the immunosuppressive tumor microenvironment, full of immunosuppressive cells, which is consistent with the poor prognosis of the high-risk group.

Then, TIMER was applied to evaluate the correlation between the expression levels of *EFNA3* and *SERPINE1* with tumor purity and infiltrating levels of immune cells (**Figures S2A, B**). It showed a correlation between immune cell infiltration and the expression levels of *EFNA3* and *SERPINE1*. It showed that the risk gene *SPERPINE1* had a significant positive correlation with the infiltration of macrophages, neutrophils and dendritic cells, and a significant negative correlation with tumor purity and B cells (*P* < 0.05, **Figure S2A**). However, the prognostic protective gene *EFNA3* showed the opposite trend for most aspects except B cells (**Figure S2B**, all *P* < 0.05).

### The Correlation Between Risk Score and Immune Checkpoint Molecules

**Figure 5:**
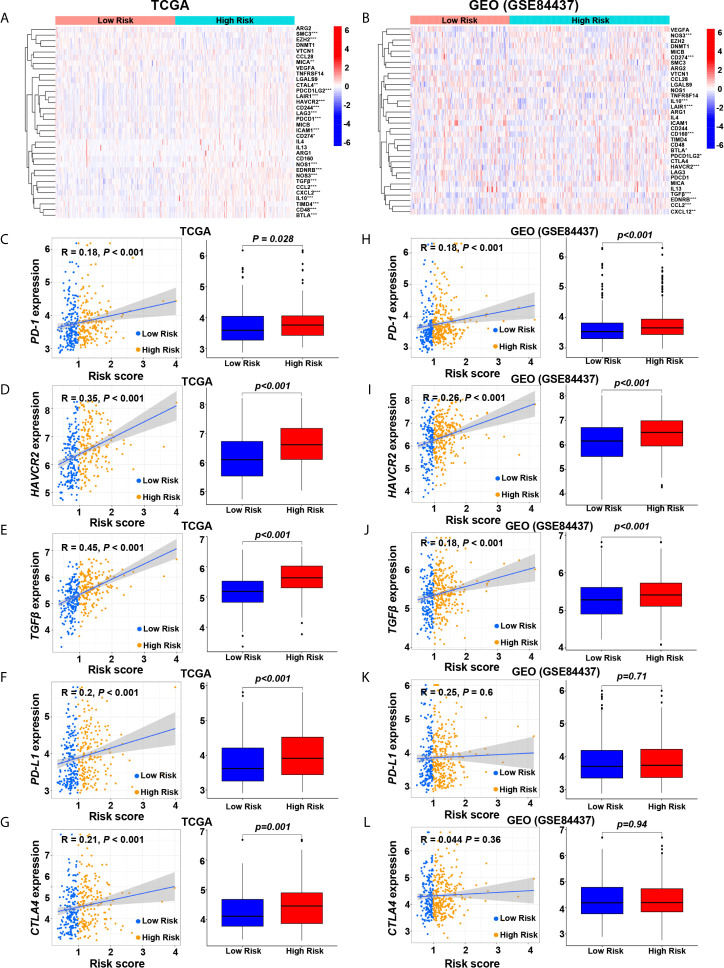
Relationships between hypoxia risk score and immune checkpoint molecules. **(A, G)** Heatmaps show the expression level of immune checkpoint molecules in high-risk and low-risk groups in the screening and validation cohorts (*P < 0.05; **P < 0.01; ***P < 0.001). Scatter plots and box plots show the relationship between the risk score and the expression level of **(B, H)** *PD-1*, **(C, I)** *HAVCR2*, **(D, J)** *TGF-β*, **(E, K)** *PD-L1*, and **(F, L)** *CTLA4* in the screening and validation cohorts.

We compared the immune checkpoint molecules between the high-risk and low-risk groups. The expression levels of many immune checkpoint molecules were higher in the high-risk group than those in the low-risk group (**Figures 5A, B**). In the screening cohort, the expression level of five key immune checkpoint molecules (*PD-1*, *PD-L1*, *CTLA-4*, *HAVCR2* and *TGF-β*) in the high-risk group was significantly higher than those in the low-risk group, and significantly positively correlated with risk score (**Figures 5C–G**). Similar results were obtained in the validation cohort, except that *CTLA4* and *PD-L1* showed no significant difference between the high-risk and low-risk groups (**Figures 5H–L**).

### The Correlation Between Risk Score and Tumor Mutation Burden and Somatic Mutation

**Figure 6:**
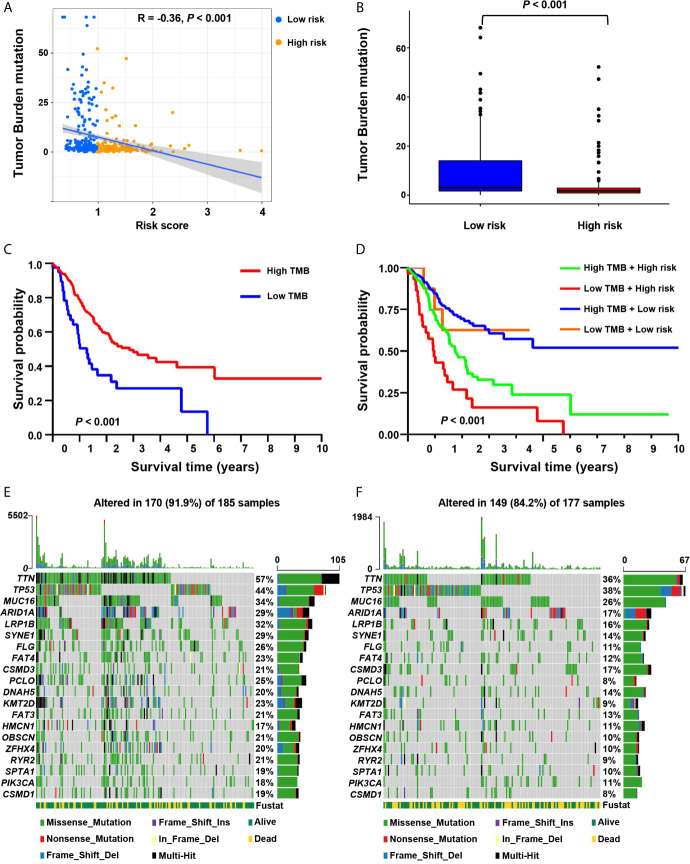
The correlation between the risk score and somatic variants. **(A)** The scatter plot depicts the negative correlation between risk score and tumor mutation burden (TMB) in the screening cohort. **(B)** TMB difference in the high-risk and low-risk groups. **(C)** Kaplan-Meier curves for high-risk and low-TMB groups of the screening cohort. **(D)** Kaplan-Meier curves for patients in the screening cohort stratified by both risk score and TMB. **(E, F)** Waterfall plots display the frequently mutated genes in low-risk and high-risk groups in the screening cohort. The left panel shows the genes ordered by their mutation frequencies. The right panel presents different mutation types.

It showed that the risk score was significantly negatively correlated with tumor mutation burden **(**TMB) (R = –0.36, *P* < 0.001; **Figure 6A**). We further compared the TMB of patients in the low-risk and high-risk groups. It showed that the TMB of the low-risk group was significantly higher than that of the high-risk group (Wilcoxon test *P* < 0.001) (**Figure 6B**). We determined the optimal cutoff value of TMB (cutoff value = 0.68) by using the minimum *P*-value method, and divided the patients into a high TMB group (n = 320) and a low TMB group (n = 42). It showed that patients in the high TMB group had a better survival prognosis than those in the low TMB group (log-rank test, *P* < 0.001, **Figure 6C**). We further evaluated the synergistic effect of the TMB grouping and the risk score grouping in the prognostic stratification. It showed that TMB status did not affect the survival prognosis prediction based on the risk score group. The risk score subgroup indicated significant survival differences in both the low and high TMB subgroups (log rank test, high TMB & high-risk *vs*. high TMB & low-risk, *P* < 0.001; low TMB & low-risk *vs*. low TMB & low-risk, *P* < 0.001; **Figure 6D**). Moreover, the high TMB & low-risk group had the best overall survival rate, and the low TMB & high-risk group had the worst overall survival rate.

Furthermore, we estimated somatic variations in gastric cancer driver genes between the low-risk and high-risk subgroups. We used Maftools to access gastric cancer driver genes and further analyzed the top 20 ones with the highest mutation frequency (**Figures 6E, F**). The results showed that there were significant differences in the mutation frequency of *PCLO*, *TTN*, *FLG*, *LRP1B*, *KMT2D*, *SYNE1*, *RYR2*, *OBSCN*, *CSMD1*, *FAT3*, *ARID1A*, *ZFHX4*, *FAT4* and *SPTA1* in the high-risk and low-risk groups (Chi-square test, all *P* < 0.05; **Table S5**).

### The Correlation Between Risk Score and Chemotherapeutic Drugs

**Figure 7:**
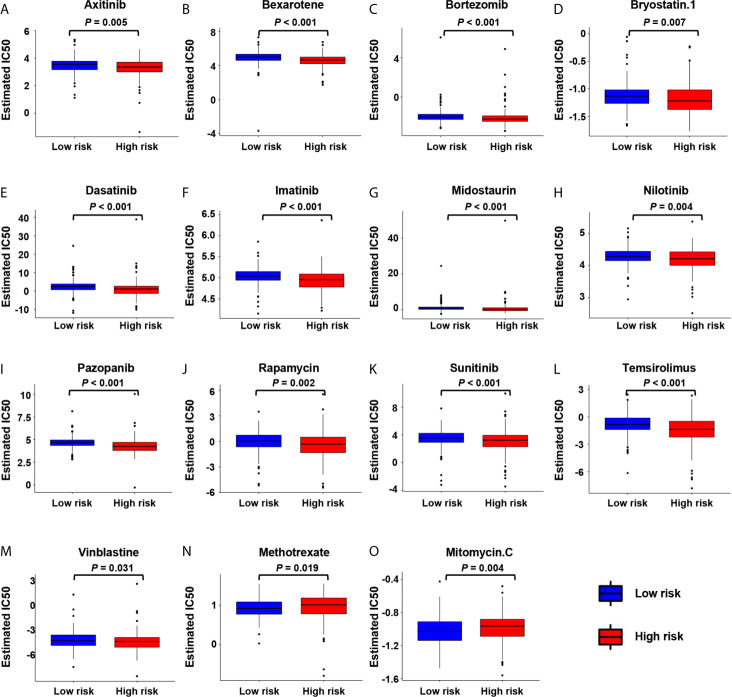
The correlation between low-risk and high-risk groups and chemotherapeutics. Sensitivity to chemotherapeutic drugs is expressed by the half inhibitory centration (IC50) of chemotherapeutic drugs. **(A)** Axitinib; **(B)** Bexarotene; **(C)** Bortezomib; **(D)** Bryostatin.1; **(E)** Dasatinib; **(F)** Imatinib; **(G)** Midostaurin; **(H)** Nilotinib; **(I)** Pazopanib; **(J)** Rapamycin; **(K)** Sunitinib; **(L)** Temsirolimus; **(M)** Vinblastine; **(N)** Methotrexate; **(O)** Mitomycin.C.

We further analyzed the association between the risk score and the efficacy of chemotherapy in the treatment of gastric cancer. It showed that the high-risk group was associated with lower half inhibitory centration (IC50) of chemotherapeutic drugs, such as axitinib (*P* = 0.0053), bexarotene (*P* < 0.001), bortezomib (*P* < 0.001), bryostatin.1 (*P* = 0.0067), dasatinib (*P* < 0.001), imatinib (*P* < 0.001), midostaurin (*P* < 0.001), nilotinib (*P* = 0.04), pazopanib (*P* = 0.0024), sunitinib (*P* < 0.001), temsirolimus (*P* < 0.001), and vinblastine (*P* = 0.031), while the IC50 of methotrexate (*P* = 0.019) and mitomycin.C (*P* = 0.0035) was higher, indicating that the risk scores can be used as a potential predictor of chemical sensitivity (**Figures 7A–O**).

### The Correlation Between Risk Score and Clinicopathological Characteristics

We conducted correlation analyses between clinicopathological factors and risk score in the screening cohort (**Figures S3C**), and tumor grade and T stage were significantly associated with risk score (**Figures S3C, D**). In the validation cohort, age, T stage, and N stage were significantly associated with risk score (**Figures S3H, J, K**).

### Development of Nomograms to Predict Individual Survival Outcomes

**Figure 8:**
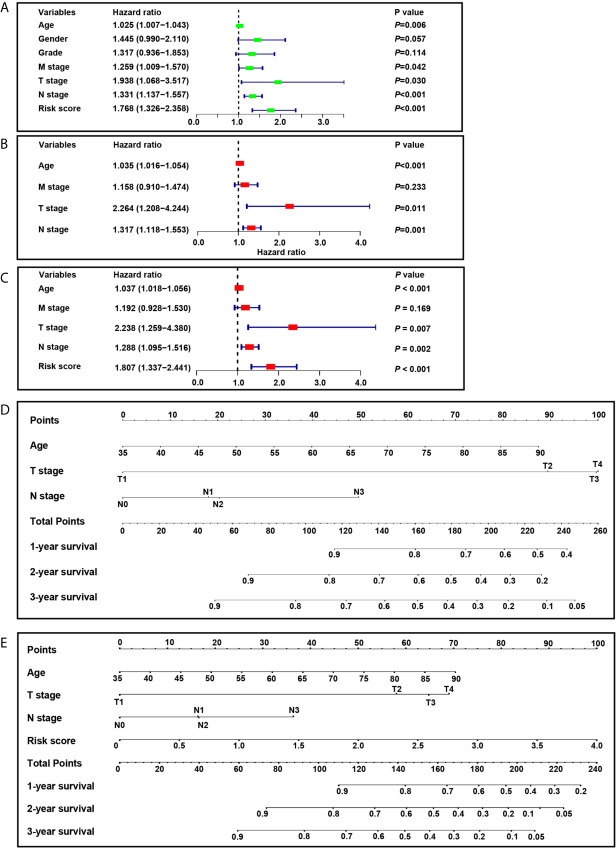
Construction of nomograms. **(A)** Univariate analysis included risk score, age, gender, grade, M stage, T stage and N stage in the screening cohort. **(B)** Cox proportional hazards analysis included age, M stage, T stage and N stage in the screening cohort. **(C)** Cox proportional hazards analysis included risk score, age, M stage, T stage and N stage in the screening cohort. **(D)** Nomogram 1 based on the clinicopathological characteristics. **(E)** Nomogram 2 based on the risk score and clinicopathological characteristics.

We developed nomograms based on the screening cohort and further verified their predictive ability in the validation cohort. It showed that age, T stage, N stage, M stage, and risk score are significant prognostic factors (**Figure 8A**). In the first step Cox proportional hazards analysis, we incorporated age, T stage, N stage, and M stage. It showed that age, T stage, and N stage were independent prognostic factors (**Figure 8B**) and were used to construct nomogram 1 (**Figure 8D**). In the second step Cox proportional hazards analysis, we incorporated age, T stage, N stage, M stage and risk score. It showed that age, T stage, N stage and risk score were independent prognostic factors (**Figure 8C**) and were used to construct nomogram 2 (**Figure 8E**).

### Comparison of Prognostic Performance and Clinical Usefulness Between Nomogram 1 and Nomogram 2

**Figure 9:**
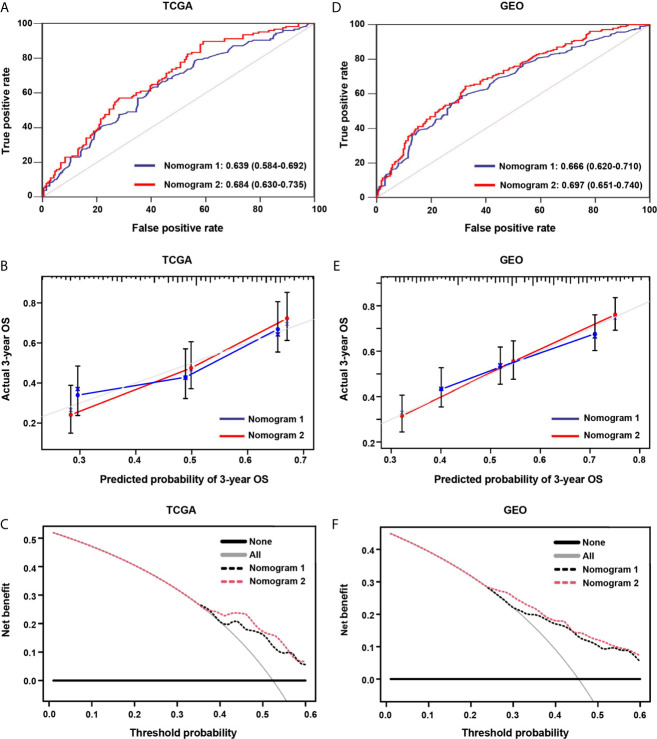
The areas under the curve (AUC), calibration curve and decision curve analysis (DCA) for predicting patient survival. **(A, D)** The AUCs assess the accuracy of the nomograms in the screening and validation cohorts. **(B, E)** The calibration curves assess the consistency of the nomograms in the screening and validation cohorts. **(C, F)** DCAs assess the clinical usefulness of nomograms in the screening and validation cohorts.

In the screening cohort, nomogram 2 showed superior prognostic ability [AUC 0.684, 95% confidence interval (CI), 0.630–0.735] compared with nomogram 1 (AUC 0.639, 95% CI, 0.584–0.692) (**Figure 9A**). The calibration curves of nomogram 2 at 3 years also showed better consistency between the predicted and observed survivals than that of nomogram 1 (**Figure 9B**). Nomogram 2 showed higher net benefit than nomogram 1 between the threshold probabilities of around 37–60% in predicting 3-year overall survival (**Figure 9C**). Similar results were found in the independent validation cohort (**Figures 9D–F**).

### Expression Levels of SERPINE1 and EFNA3 in GC Cell Lines and Tissues

**Figure 10:**
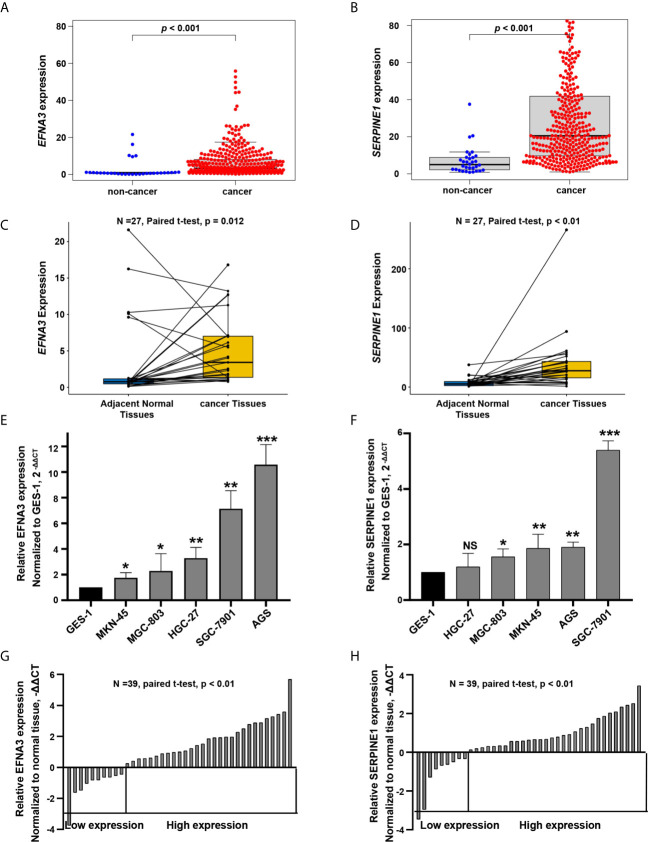
*EFNA3* and *SERPINE1* are upregulated in gastric cancer cell lines and tissues. **(A, B)** Bioinformatics analysis of the expression of *EFNA3* and *SERPINE1* in cancer and non-cancerous tissues in TCGA. **(C, D)** Bioinformatics analysis of the expression of *EFNA3* and *SERPINE1* in 27 pairs of gastric cancer and adjacent non-cancerous tissues in TCGA. **(E, F)** qRT-PCR results of *EFNA3* and *SERPINE1* expression level in GES-1 and gastric cancer cell lines. (Data are presented as mean ± SD. NS: P ≥ 0.05, *P < 0.05, **P < 0.01, ***P < 0.001). **(G, H)** qRT-PCR results of *EFNA3* and *SERPINE1* expression level in 39 pairs of gastric cancer and adjacent non-cancerous tissues. (Data are shown as –ΔΔCT values).

In the screening cohort, the expression of *SERPINE1* and *EFNA3* in tumor tissues was up-regulated when compared with adjacent non-cancerous tissues and normal tissues (*P* < 0.05, **Figures 10A–D**). To confirm the expression levels of *SERPINE1* and *EFNA3* in gastric cancer, we subsequently verified it in gastric cancer cell lines and patient tissues by qRT-PCR experiments. The results showed that, when compared with gastric normal epithelial mucosae cell line GES-1 and adjacent non-cancerous tissues, the expression of *SERPINE1* and *EFNA3* were significantly higher in gastric cancer cell lines (**Figures 10E, F**, except for *SERPINE1* in HGC-27, *P* > 0.05) and gastric cancer tissues (*P* < 0.05) (**Figures 10G, H**).

## Discussion

Hypoxia is caused by an imbalance between insufficient oxygen supply and increased oxygen demand (21, 33). It is also one significant characteristic of tumor microenvironment. Tumor cells adapt to and rely on tumor microenvironment, contributing to instability and diversity of gene mutations, and activating a variety of signaling pathways and cytokines, contributing to the angiogenesis, invasion, metastasis, epithelial-mesenchymal transition, cancer stem cell maintenance, immune escape and resistance to radiotherapy and chemotherapy (34, 35). Therefore, understanding the molecular mechanism of hypoxia is critical to improving the survival of cancer therapy.

In this study, we identified two prognosis-related hypoxia genes, *SERPINE1* and *EFNA3*, and establish a hypoxia risk score model based on the two genes. Subsequent survival analysis indicated that the high-risk group was associated with poorer prognosis, which was verified by an independent GEO cohort. GSEA analysis showed that the high-risk group was significantly enriched in pathways for tumor progression, such as the JAK-STAT signaling pathway (36), cancer in pathway, TGF-β signaling pathway (37, 38), and NOTCH signaling pathway (39), leading to poor prognosis. In the hypoxic microenvironment, HIFs are the main regulators of hypoxic response (18, 35). HIFs can cause the malignant phenotype of tumors by activating or enhancing JAK-STAT signaling pathway, TGF-β signaling pathway and NOTCH signaling pathway (40–43). Besides, the TCGA data base and qRT-PCR analysis confirmed the overexpression of these two hypoxia genes in tumor tissues and gastric cancer cell lines when compared with normal tissues and gastric normal epithelial cell line. Finally, the risk score model, age, T stage, and N stage were identified as independent risk factors related to OS and included in the nomogram. It showed that the nomogram was an effective tool for predicting the prognosis. The two-gene signature has a powerful ability to predict the prognosis of patients with gastric cancer, and may be helpful to guide clinical treatment decisions.

Tumor purity can reflect the characteristics of the tumor microenvironment. The risk score was significantly positively correlated with infiltrating immune cells and stromal cells, but negatively correlated with tumor purity. Previous studies showed that low tumor purity is associated with poor prognosis of multiple tumor types (44–46). We speculated that low-purity tumors may recruit more tumor immunosuppressive cells than high-purity tumors, and further studied the relationship between the risk score and the subtypes of infiltrating immune cells. We found that the tumors in the high-risk group contained more infiltrating immunosuppressive cells such as Tregs, macrophages, neutrophils, para-inflammatory and mast cells than the low-risk group. A previous study found that Tregs suppressed the anti-tumor immune response by weakening the cell-mediated immune response to tumors, thereby promoting disease progression (47). Hypoxia can protect tumors from the intrinsic anti-tumor immune response by forming an immunosuppressive microenvironment, which may explain a poor prognosis of the high-risk group.

Cytokine are important factors in regulating tumor immunity. Among them, tumor immunosuppressive cytokines are important factors inhibiting immune cell activity. Transforming growth factor-β (TGF-β) suppresses the immune system by inhibiting the maturation of dendritic cells, inhibiting the activity of NK cells, and reducing the cytotoxicity of T cells (47, 48). Interleukin 10 (IL-10) is an immunosuppressive cytokine secreted by T-helper 2 (Th2) cells, Tregs, and M2 macrophages. It has been shown to impair the proliferation, cytokine production and migration capabilities of effector T cells (49). IL-10 also promotes the stable expression of Foxp3, TGF-β-receptor 2 and TGF-β, thereby stabilizing the phenotype and functions of Treg (50). In our research, the immunosuppressive cytokines, such as IL-10 and TGF-β, were up-regulated in the high-risk group, thereby further promoting immunosuppression.

The correlation between the intrinsic escape mechanism and risk score is clinically important. The inherent immune escape of tumors demonstrates that tumor cells can mediate their own immune escape directly. Previous study has illustrated that the expression of immune check-point molecules and tumor immunogenicity are two important aspects of intrinsic immune escape (51). Immune checkpoint molecules play a key role in tumor progression and carcinogenesis by promoting tumor immunosuppression. Malignant tumors can evade immune killing by stimulating immune checkpoint target genes (such as *PD-1*, *PD-L1*, *CTLA-4*, *TGF-β*, and *HAVCR2*). In this study, immune checkpoint molecules of *PD-1*, *TGF-β*, and *HAVCR2* were up-regulated in the high-risk group. This result indicates that tumor cells in the high-risk group express immune checkpoint molecules to protect themselves from attack.

Another potentially significant intrinsic immune escape mechanism is immunogenicity. Some somatic mutations in tumor DNA produce neoantigens, and the antigens from this mutation are recognized and targeted by the immune system, especially after treatment with drugs that activate T cells (52–56). The more somatic mutations are present in a tumor, the more neoantigens it may form. TMB may represent a better estimate of tumor neoantigen burden (57). Here, we found that the high-risk group had a lower proportion of somatic mutations, and the hypoxia risk score was significantly negatively correlated with TMB. Tumor cells in the hyperoxia group produced fewer neoantigens, thus avoiding being recognized and killed by T cells.

To investigate the role of hypoxia risk in drug treatment, our research showed that, the tumors in the high-risk group were not sensitive to most chemotherapy drugs, such as axitinib, bexarotene, bortezomib, and imatinib. However, the tumors in the high-risk group were more sensitive to methotrexate and mitomycin.c and may benefit from these two chemotherapy drugs. Those in the high-risk group express higher levels of *PD-1*, *HAVCR2* and other immune checkpoint molecules to avoid the attack of anti-tumor immune cells. The high-risk group may benefit from immunotherapy, such as the use of *PD-1* and *HAVCR2* inhibitors.

Nomograms are commonly used to assess the prognosis of tumors (58, 59). In this study, we constructed two prognostic nomograms. Nomogram 1 is based on clinical characteristics, and nomogram 2 is developed by the combination of clinical characteristics and the hypoxia risk score model. It showed that the prognostic nomogram based on the combination of clinical characteristics and hypoxia risk score model has better predictive ability and higher clinical usefulness. However, owing to the lack of *in vitro* or *in vivo* experiments, the reliability of our molecular mechanism analysis may be limited.

## Conclusions

In summary, we developed and validated a hypoxia risk score model based on a novel hypoxia-related gene signature revealing the relationship between hypoxia and tumor immune microenvironment. The current study may provide new insights into how hypoxia affects the prognosis, and may be helpful in guiding targeted hypoxia therapy for gastric cancer.

## Data Availability Statement

The datasets analyzed during this current study are available in TCGA (http://cancergenome.nih.gov/) and GEO (https://www.ncbi.nlm.nih.gov/geo, GSE84437) data bases. The data are also available from the corresponding author (zhangchundong2007@126.com or daidq63@163.com) on reasonable request.

## Ethics Statement

The studies involving human participants were reviewed and approved by Ethics Committee of the Medical Association of the Fourth Affiliated Hospital of China Medical University (EC-2021-KS-043). The patients/participants provided their written informed consent to participate in this study.

## Author Contributions

C-DZ and D-QD have contributed equally to this work as co-corresponding authors. J-PP and C-DZ wrote the main text and performed data analysis. J-PP, C-DZ, and D-QD designed the study and collected the data. All authors contributed to the article and approved the submitted version.

## Funding

This research was funded in part by the China Scholarship Council (201908050148).

## Conflict of Interest

The authors declare that the research was conducted in the absence of any commercial or financial relationships that could be construed as a potential conflict of interest.

## References

[1] Sung H Ferlay J L Siegel R Laversanne M Soerjomataram I Jemal A. Global Cancer Statistics 2020: GLOBOCAN Estimates of Incidence and Mortality Worldwide for 36 Cancers in 185 Countries. CA: Cancer J Clin (2021) 71(3):209–49. 10.3322/caac.21660 33538338

[2] Egner JR. AJCC Cancer Staging Manual. JAMA J Am Med Assoc (2010) 304(15):1726–7. 10.1001/jama.2010.1525

[3] Ajani JA D’Amico TA Almhanna K Bentrem DJ Chao J Das P. Gastric Cancer, Version 3.2016, NCCN Clinical Practice Guidelines in Oncology. J Natl Compr Cancer Netw JNCCN (2016) 14(10):1286. 10.6004/jnccn.2016.0137 27697982

[4] Shah MA Ajani JA. Gastric Cancer—An Enigmatic and Heterogeneous Disease. JAMA J Am Med Assoc (2010) 303(17):1753–4. 10.1001/jama.2010.553 20442394

[5] Noh SH Park SR Yang HK Chung HC Chung IJ Kim SW. Adjuvant Capecitabine Plus Oxaliplatin for Gastric Cancer After D2 Gastrectomy (CLASSIC): 5-Year Follow-Up of an Open-Label, Randomised Phase 3 Trial. Lancet Oncol (2014) 15(12):1389–96. 10.1016/S1470-2045(14)70473-5 25439693

[6] Khouzam RA Goutham HV Zaarour RF Chamseddine AN Chouaib S. Integrating Tumor Hypoxic Stress in Novel and More Adaptable Strategies for Cancer Immunotherapy. Semin Cancer Biol (2020) 65:140–54. 10.1016/j.semcancer.2020.01.003 31927131

[7] PaiSheng C WenTai C PeiLing H ShihChieh L IChen P ChiaYih W. Pathophysiological Implications of Hypoxia in Human Diseases. J Biomed Sci (2020) 27(1):63. 10.1186/s12929-020-00658-7 32389123 PMC7212687

[8] Vaupel P Kelleher D Thews O. Modulation of Tumor Oxygenation. Int J Radiat Oncol Biol Phys (1998) 42(4):843–8. 10.1016/S0360-3016(98)00324-1 9845108

[9] Petrova V Annicchiarico-Petruzzelli M Melino G Amelio I. The Hypoxic Tumour Microenvironment. Oncogenesis (2018) 7(1):10. 10.1038/s41389-017-0011-9 29362402 PMC5833859

[10] Barbara M Pilar D Feda A Azab AK. The Role of Hypoxia in Cancer Progression, Angiogenesis, Metastasis, and Resistance to Therapy. Hypoxia (2015) 3:83–92. 10.2147/HP.S93413 27774485 PMC5045092

[11] Shida M Kitajima Y Nakamura J Yanagihara K Baba K Wakiyama K. Impaired Mitophagy Activates mtROS/HIF-1 Alpha Interplay and Increases Cancer Aggressiveness in Gastric Cancer Cells Under Hypoxia. Int J Oncol (2016) 48(4):1379–90. 10.3892/ijo.2016.3359 26820502

[12] Gilkes DM Semenza GL Wirtz D. Hypoxia and the Extracellular Matrix: Drivers of Tumour Metastasis. Nat Rev Cancer (2014) 14(6):430. 10.1038/nrc3726 24827502 PMC4283800

[13] Barsoum IB Smallwood CA Siemens DR Graham CH. A Mechanism of Hypoxia-Mediated Escape From Adaptive Immunity in Cancer Cells. Cancer Res (2014) 74(3):665–74. 10.1158/0008-5472.CAN-13-0992 24336068

[14] Wang H Zhao L Zhu LT Wang Y Pan D Yao J. Wogonin Reverses Hypoxia Resistance of Human Colon Cancer HCT116 Cells *Via* Downregulation of HIF-1α and Glycolysis, by Inhibiting PI3K/Akt Signaling Pathway. Mol Carcinog (2014) 53(S1):E107–E18. 10.1002/mc.22052 23761018

[15] Mucaj V Shay J Simon MC. Effects of Hypoxia and HIFs on Cancer Metabolism. Int J Hematol (2012) 95(5):464–70. 10.1007/s12185-012-1070-5 22539362

[16] Takahashi R Tanaka S Hiyama T Ito M Kitadai Y Sumii M. Hypoxia-Inducible Factor-1 Alpha Expression and Angiogenesis in Gastrointestinal Stromal Tumor of the Stomach. Oncol Rep (2003) 10(4):797–802. 10.3892/or.10.4.797 12792726

[17] Chen WT Huang CJ Wu MT Yang SF Su YC Chai CY. Hypoxia-Inducible Factor-1α Is Associated With Risk of Aggressive Behavior and Tumor Angiogenesis in Gastrointestinal Stromal Tumor. JPN J Clin Oncol (2005) 35(4):207–13. 10.1093/jjco/hyi067 15845570

[18] Zhang J Xu J Dong Y Bo H. Down-Regulation of HIF-1α Inhibits the Proliferation, Migration, and Invasion of Gastric Cancer by Inhibiting PI3K/AKT Pathway and VEGF Expression. Biosci Rep (2018) 38(6):BSR20180741. 10.1042/BSR20180741 29899167 PMC6435555

[19] Hao LS Liu Q Tian C Zhang DX Yuan ZX. Correlation and Expression Analysis of Hypoxiainducible Factor1α, Glucose Transporter 1 and Lactate Dehydrogenase5 in Human Gastric Cancer. Oncol Lett (2019) 18(2):1431–41. 10.3892/ol.2019.10457 PMC6607088 31423208

[20] Hanahan D Coussens L. Accessories to the Crime: Functions of Cells Recruited to the Tumor Microenvironment. Cancer Cell (2012) 21(3):309–22. 10.1016/j.ccr.2012.02.022 22439926

[21] Hanahan D Weinberg RA. The Hallmarks of Cancer. Cell (2000) 100(1):57–70. 10.1016/S0092-8674(00)81683-9 10647931

[22] Lazăr D Avram MF Romoșan I Cornianu M Tăban S Goldiș A. Prognostic Significance of Tumor Immune Microenvironment and Immunotherapy: Novel Insights and Future Perspectives in Gastric Cancer. World J Gastroenterol (2018) 24(32):3583–616. 10.3748/wjg.v24.i32.3583 PMC6113718 30166856

[23] Lin W Wu S Chen X Ye Y Qiu S. Characterization of Hypoxia Signature to Evaluate the Tumor Immune Microenvironment and Predict Prognosis in Glioma Groups. Front Oncol (2020) 10:796. 10.3389/fonc.2020.00796 32500034 PMC7243125

[24] Zhang B Tang B Gao J Li J Qin L. A Hypoxia-Related Signature for Clinically Predicting Diagnosis, Prognosis and Immune Microenvironment of Hepatocellular Carcinoma Patients. J Trans Med (2020) 18(1):342. 10.21203/rs.3.rs-17783/v3 PMC7487492 32887635

[25] Yoon SJ Park J Shin Y Choi Y Huh YM. Deconvolution of Diffuse Gastric Cancer and the Suppression of CD34 on the BALB/c Nude Mice Model. BMC Cancer (2020) 20(1):314. 10.1186/s12885-020-06814-4 32293340 PMC7160933

[26] Tibshirani R. The LASSO Method for Variable Selection in the Cox Model. Stat Med (1997) 16(4):385–95. 10.1002/(SICI)1097-0258(19970228)16:4<385::AID-SIM380>3.0.CO;2-3 9044528

[27] Yoshihara K Shahmoradgoli M Martínez E Vegesna R Kim H Torres-Garcia W. Inferring Tumour Purity and Stromal and Immune Cell Admixture From Expression Data. Nat Commun (2013) 4:2612. 10.1038/ncomms3612 24113773 PMC3826632

[28] Taiwen L Jingxin F Zexian Z David C Jing L Qianming C. TIMER2.0 for Analysis of Tumor-Infiltrating Immune Cells. Nucleic Acids Res (2020) 48(W1):W509–W14. 10.1093/nar/gkaa407 PMC7319575 32442275

[29] Subramanian A Tamayo P Mootha VK Mukherjee S Ebert BL Gillette MA. Gene Set Enrichment Analysis: A Knowledge-Based Approach for Interpreting Genome-Wide Expression Profiles. Proc Natl Acad Sci USA (2005) 102(43):P.15545–50. 10.1073/pnas.0506580102 PMC1239896 16199517

[30] Pages F Mlecnik B Marliot F Bindea G Ou FS Bifulco C. International Validation of the Consensus Immunoscore for the Classification of Colon Cancer: A Prognostic and Accuracy Study. Lancet (2018) 391(10135):2128–39. 10.1016/S0140-6736(18)30789-X 29754777

[31] Fitzgerald M Saville BR Lewis RJ. Decision Curve Analysis. JAMA (2015) 313(4):409–10. 10.1001/jama.2015.37 25626037

[32] Vickers AJ Elkin EB. Decision Curve Analysis: A Novel Method for Evaluating Prediction Models. Med Decision Making (2006) 26(6):565–74. 10.1177/0272989X06295361 PMC2577036 17099194

[33] Brahimi-Horn MC Chiche J Pouysségur J. Hypoxia and Cancer. J Mol Med (2007) 85(12):1301–7. 10.1007/s00109-007-0281-3 18026916

[34] Rosella FA Lucia C Michela S Veronica Z Stefano G Reay MA. Hypoxia-Induced Alternative Splicing: The 11th Hallmark of Cancer. J Exp Clin Cancer Res: CR (2020) 39(1):110. 10.1186/s13046-020-01616-9 32536347 PMC7294618

[35] Schito L Semenza GL. Hypoxia-Inducible Factors: Master Regulators of Cancer Progression. Trends Cancer (2016) 2(12):758–70. 10.1016/j.trecan.2016.10.016 28741521

[36] Owen KL Brockwell NK Parker BS. Jak-STAT Signaling: A Double-Edged Sword of Immune Regulation and Cancer Progression. Cancers (2019) 11(12):2002. 10.3390/cancers11122002 PMC6966445 31842362

[37] Yang L Pang Y Moses HL. TGF-Beta and Immune Cells: An Important Regulatory Axis in the Tumor Microenvironment and Progression. Trends Immunol (2010) 31(6):220–7. 10.1016/j.it.2010.04.002 PMC2891151 20538542

[38] Ming LS Ming O. Tgf-β Control of Adaptive Immune Tolerance: A Break From Treg Cells. BioEssays (2018) 40(11):e1800063. 10.1002/bies.201800063 30159904 PMC6300063

[39] Goruganthu MUL Shanker A Dikov MM Carbone DP. Specific Targeting of Notch Ligand-Receptor Interactions to Modulate Immune Responses: A Review of Clinical and Preclinical Findings. Front Immunol (2020) 11:1958. 10.3389/fimmu.2020.01958 32922403 PMC7456812

[40] Tian Q Yan X Zheng W Sun R Ji W Wang X. Overexpression of Hypoxia-Inducible Factor 1α Induces Migration and Invasion Through Notch Signaling. Int J Oncol (2015) 47(2):728–38. 10.3892/ijo.2015.3056 26094772

[41] Chen J Imanaka N Chen J Griffin JD. Hypoxia Potentiates Notch Signaling in Breast Cancer Leading to Decreased E-cadherin Expression and Increased Cell Migration and Invasion. Br J Cancer (2010) 102(2):351–60. 10.1038/sj.bjc.6605486 PMC2816657 20010940

[42] Tirpe AA Gulei D Ciortea SM Crivii C Berindan-Neagoe I. Hypoxia: Overview on Hypoxia-Mediated Mechanisms With a Focus on the Role of HIF Genes. Int J Mol Sci (2019) 20(24):6140. 10.3390/ijms20246140 PMC6941045 31817513

[43] Young LM Hee JY Joung LE Jong-Hwan P Sang-Kyu Y Taekyu P. Phosphorylation and Activation of STAT Proteins by Hypoxia in Breast Cancer Cells. Breast (2006) 15(2):187–95. 10.1016/j.breast.2005.05.005 16084091

[44] Gong Z Zhang J Guo W. Tumor Purity as a Prognosis and Immunotherapy Relevant Feature in Gastric Cancer. Cancer Med (2020) 9(23):9052–63. 10.1002/cam4.3505 PMC7724479 33030278

[45] Zhang C Cheng W Ren X Wang Z Liu X Li G. Tumor Purity as an Underlying Key Factor in Glioma. Clin Cancer Res (2017) 23(20):6279–91. 10.1158/1078-0432.CCR-16-2598 28754819

[46] Mao Y Feng Q Zheng P Yang L Liu T Xu Y. Low Tumor Purity Is Associated With Poor Prognosis, Heavy Mutation Burden, and Intense Immune Phenotype in Colon Cancer. Cancer Manage Res (2018) 10:2569–3577. 10.2147/CMAR.S171855 PMC6149864 30271205

[47] Nishikawa H Sakaguchi S. Regulatory T Cells in Cancer Immunotherapy. Ann New York Acad Sci (2014) 27(1):1–7. 10.1016/j.coi.2013.12.005 24413387

[48] Haque S Morris JC. Transforming Growth Factor-β: A Therapeutic Target for Cancer. Hum Vaccines Immunotherapeutics (2017) 13(8):1741–50. 10.1080/21645515.2017.1327107 PMC5557219 28575585

[49] Dennis KL Blatner NR Gounari F Khazaie K. Current Status of Interleukin-10 and Regulatory T-Cells in Cancer. Curr Opin Oncol (2013) 25(6):637–45. 10.1097/CCO.0000000000000006 PMC4322764 24076584

[50] Barbi J Pardoll D Pan F. Treg Functional Stability and Its Responsiveness to the Microenvironment. Immunol Rev (2014) 259(1):115–39. 10.1111/imr.12172 PMC3996455 24712463

[51] Schreiber RD Old LJ Smyth MJ. Cancer Immunoediting: Integrating Immunity’s Roles in Cancer Supression and Promotion. Science (2011) 331(6024):1565–70. 10.1126/science.1203486 21436444

[52] Snyder A Makarov V Merghoub T Yuan J Zaretsky J Desrichard A. Genetic Basis for Clinical Response to CTLA-4 Blockade in Melanoma. N Engl J Med (2014) 372(23):2189–99. 10.1056/NEJMoa1406498 PMC4315319 25409260

[53] Matsushita H Vesely M Koboldt D Rickert C Uppaluri R Magrini V. Cancer Exome Analysis Reveals a T-Cell-Dependent Mechanism of Cancer Immunoediting. Nature (2012) 482(7385):400–4. 10.1038/nature10755 PMC3874809 22318521

[54] Riaz N Morris L Havel J Makarov V Desrichard A Chan T. The Role of Neoantigens in Response to Immune Checkpoint Blockade. Int Immunol (2016) 28(8):411–9. 10.1093/intimm/dxw019 PMC4986233 27048318

[55] Ott PA Zhuting H Keskin DB Shukla SA Jing S Bozym DJ. An Immunogenic Personal Neoantigen Vaccine for Patients With Melanoma. Nature (2018) 547(7662):217–21. 10.1038/nature22991 PMC5577644 28678778

[56] Cohen CJ Gartner JJ Horovitz-Fried M Shamalov K Robbins PF. Isolation of Neoantigen-Specific T Cells From Tumor and Peripheral Lymphocytes. J Clin Invest (2015) 125(10):3981–91. 10.1172/JCI82416 PMC4607110 26389673

[57] Chan TA Yarchoan M Jaffee E Swanton C Quezada SA Stenzinger A. Development of Tumor Mutation Burden as an Immunotherapy Biomarker: Utility for the Oncology Clinic. Ann Oncol (2019) 30(1):44–56. 10.1093/annonc/mdy495 30395155 PMC6336005

[58] Pei JP Zhang CD Liang Y Zhang C Dai DQ. Novel Nomograms Individually Predicting Overall Survival of Non-Metastatic Colon Cancer Patients. Front Oncol (2020) 10:733. 10.3389/fonc.2020.00733 32435623 PMC7218119

[59] Alexia I Deborah S V RG S PK. How to Build and Interpret a Nomogram for Cancer Prognosis. J Clin Oncol (2008) 26(8):1364–70. 10.1200/JCO.2007.12.9791 18323559

