# Supplementary material for: Screening and Validation of the Hypoxia-Related Signature of Evaluating Tumor Immune Microenvironment and Predicting Prognosis in Gastric Cancer
Source: Front Immunol. 2021 Jun 25;12:705511. doi: 10.3389/fimmu.2021.705511 (PMC8267919; doi:10.3389/fimmu.2021.705511)
Supplement: Supplementary file 1 [file DataSheet_1.docx]

Supplementary Material

| Table S1. All primers of qRT-PCR used in this study. | |
| --- | --- |
| Gene | Sequence (5’-3’) |
| SERPINE1-F | TCGAGGTGAACGAGAGTGGCA |
| SERPINE1-R | AAGGACTGTTCCTGTGGGGTTGT |
| EFNA3-F | CCTTCTCTCTGGGCTACGAGTTCC |
| EFNA3-R | GCAGACGAACACCTTCATCCTCAG |
| GAPDH-F | AGCCACATCGCTCAGACTC |
| GAPDH-R | GCCCAATACGACCAAATTC |

| Table S2. Baseline characteristics of gastric cancer patients in the screening and validation cohorts. | | | | | | |
| --- | --- | --- | --- | --- | --- | --- |
| variables | TCGA | |  | GEO | | |
|  | Number | % |  | Number | % |  |
| Age |  |  |  |  |  | |
| ≤ 65 | 167 | 45 |  | 283 | 65.4 | |
| > 65 | 204 | 55 |  | 150 | 34.6 | |
| Gender |  |  |  |  |  | |
| Female | 137 | 36.9 |  | 137 | 31.6 | |
| Male | 234 | 63.1 |  | 296 | 68.4 | |
| T stage |  |  |  |  |  | |
| T1 | 17 | 4.6 |  | 11 | 2.5 | |
| T2 | 74 | 19.9 |  | 38 | 8.8 | |
| T3 | 177 | 47.7 |  | 92 | 21.2 | |
| T4 | 103 | 27.8 |  | 292 | 67.5 | |
| N stage |  |  |  |  |  | |
| N0 | 117 | 31.5 |  | 80 | 18.5 | |
| N1 | 97 | 26.1 |  | 188 | 43.4 | |
| N2 | 79 | 21.3 |  | 132 | 30.5 | |
| N3 | 78 | 21.1 |  | 33 | 7.6 | |
| Grade |  |  |  |  |  | |
| Grade I | 8 | 2.2 |  | – | – | |
| Grade II | 126 | 34 |  | – | – | |
| Grade III | 237 | 63.8 |  | – | – | |
| TNM stage |  |  |  | – | – | |
| I | 46 | 12.4 |  | – | – | |
| II | 119 | 32.1 |  | – | – | |
| III | 165 | 44.4 |  | – | – | |
| IV | 41 | 11.1 |  | – | – | |
| TCGA, The Cancer Genome Atlas; GEO, Gene Expression Omnibus. | | | | | | |

| Table S3. Differentially expressed genes related to hypoxia in gastric cancer. | | | | |
| --- | --- | --- | --- | --- |
| Gene | logFC | *FDR | Regulated | **P value |
| STC1 | 1.289 | < 0.001 | Up-Regulated | < 0.001 |
| DUSP1 | -1.518 | < 0.001 | Down-Regulated | < 0.001 |
| HK2 | 1.204 | < 0.001 | Up-Regulated | < 0.001 |
| JUN | -1.154 | 0.001 | Down-Regulated | < 0.001 |
| ZFP36 | -1.455 | < 0.001 | Down-Regulated | < 0.001 |
| FOS | -1.172 | < 0.001 | Down-Regulated | < 0.001 |
| VLDLR | -1.01 | < 0.001 | Down-Regulated | < 0.001 |
| DCN | -1.183 | < 0.001 | Down-Regulated | < 0.001 |
| EFNA1 | 1.287 | < 0.001 | Up-Regulated | < 0.001 |
| IGFBP3 | 1.576 | < 0.001 | Up-Regulated | < 0.001 |
| BGN | 2.91 | < 0.001 | Up-Regulated | < 0.001 |
| B4GALNT2 | -1.66 | 0.001 | Down-Regulated | < 0.001 |
| PIM1 | -1.161 | < 0.001 | Down-Regulated | < 0.001 |
| KIF5A | -1.054 | 0.022 | Down-Regulated | 0.014 |
| CITED2 | -1.393 | < 0.001 | Down-Regulated | < 0.001 |
| SERPINE1 | 2.399 | < 0.001 | Up-Regulated | < 0.001 |
| CAV1 | -1.175 | < 0.001 | Down-Regulated | < 0.001 |
| EFNA3 | 1.262 | < 0.001 | Up-Regulated | < 0.001 |
| STC2 | 1.991 | < 0.001 | Up-Regulated | < 0.001 |
| HOXB9 | 3.095 | < 0.001 | Up-Regulated | < 0.001 |
| PDGFB | 1.133 | < 0.001 | Up-Regulated | < 0.001 |
| PGF | 1.47 | < 0.001 | Up-Regulated | < 0.001 |
| SLC6A6 | 1.232 | < 0.001 | Up-Regulated | < 0.001 |
| P4HA1 | 1.437 | < 0.001 | Up-Regulated | < 0.001 |
| PYGM | -2.751 | < 0.001 | Down-Regulated | < 0.001 |
| DTNA | -1.011 | < 0.001 | Down-Regulated | < 0.001 |
| EDN2 | -1.919 | < 0.001 | Down-Regulated | < 0.001 |
| VEGFA | 1.261 | < 0.001 | Up-Regulated | < 0.001 |
| ALDOC | -1.111 | < 0.001 | Down-Regulated | < 0.001 |
| LDHC | 1.109 | 0.008 | Up-Regulated | 0.005 |
| PPP1R3C | -2.446 | < 0.001 | Down-Regulated | < 0.001 |
| SRPX | -1.289 | < 0.001 | Down-Regulated | < 0.001 |
| LOX | 1.822 | < 0.001 | Up-Regulated | < 0.001 |
| TKTL1 | 5.698 | 0.006 | Up-Regulated | 0.003 |
| MT1E | -1.214 | < 0.001 | Down-Regulated | < 0.001 |
| COL5A1 | 1.659 | < 0.001 | Up-Regulated | < 0.001 |
| GAPDHS | 1.026 | 0.004 | Up-Regulated | 0.002 |
| AKAP12 | -1.083 | 0.016 | Down-Regulated | 0.01 |
| CDKN1A | -1.064 | < 0.001 | Down-Regulated | < 0.001 |
| IGFBP1 | 5.519 | < 0.001 | Up-Regulated | < 0.001 |
| PLAUR | 1.149 | < 0.001 | Up-Regulated | < 0.001 |
| *Limma R package; **Log-rank test.  FC, fold change; FDR, false discovery rate. | | | | |

| Table S4. Differentially expressed genes related to the prognosis in gastric cancer. | | | |
| --- | --- | --- | --- |
| Gene | HR | 95% CI | *P value |
| AKAP12 | 1.177 | 1.042–1.329 | 0.009 |
| STC1 | 1.314 | 1.116–1.547 | 0.001 |
| CAV1 | 1.182 | 1.043–1.339 | 0.009 |
| SRPX | 1.155 | 1.018–1.31 | 0.025 |
| DCN | 1.178 | 1.04 –1.335 | 0.01 |
| IGFBP1 | 1.168 | 1.022–1.334 | 0.022 |
| EFNA3 | 0.831 | 0.726–0.952 | 0.008 |
| BGN | 1.193 | 1.046–1.36 | 0.008 |
| ZFP36 | 1.276 | 1.069–1.523 | 0.007 |
| PIM1 | 1.291 | 1.047–1.593 | 0.017 |
| COL5A1 | 1.164 | 1.014–1.337 | 0.031 |
| LOX | 1.296 | 1.109–1.513 | 0.001 |
| SERPINE1 | 1.267 | 1.135–1.415 | < 0.001 |
| DUSP1 | 1.272 | 1.102–1.469 | 0.001 |
| *Log-rank test.  HR, hazard ratio. | | | |

| Table S5. Association of risk score with Somatic Variants. | | | | | |
| --- | --- | --- | --- | --- | --- |
| gene | High-risk wild | High-risk mutation | Low-risk wild | Low-risk mutation | P value |
| TTN | 113 (63.84%) | 64 (36.16%) | 80 (43.24%) | 105 (56.76%) | < 0.001 |
| TP53 | 110 (62.15%) | 67 (37.85%) | 103 (55.68%) | 82 (44.32%) | 0.2527 |
| MUC16 | 131 (74.01%) | 46 (25.99%) | 122 (65.95%) | 63 (34.05%) | 0.1193 |
| ARID1A | 147 (83.05%) | 30 (16.95%) | 131 (70.81%) | 54 (29.19%) | 0.0084 |
| LRP1B | 149 (84.18%) | 28 (15.82%) | 126 (68.11%) | 59 (31.89%) | 0.0006 |
| SYNE1 | 153 (86.44%) | 24 (13.56%) | 132 (71.35%) | 53 (28.65%) | 0.0007 |
| FLG | 158 (89.27%) | 19 (10.73%) | 136 (73.51%) | 49 (26.49%) | 0.0002 |
| FAT4 | 155 (87.57%) | 22 (12.43%) | 142 (76.76%) | 43 (23.24%) | 0.0110 |
| CSMD3 | 147 (83.05%) | 30 (16.95%) | 147 (79.46%) | 38 (20.54%) | 0.4594 |
| PCLO | 163 (92.09%) | 14 (7.91%) | 136 (73.51%) | 49 (26.49%) | < 0.001 |
| DNAH5 | 153 (86.44%) | 24 (13.56%) | 149 (80.54%) | 36 (19.46%) | 0.1714 |
| KMT2D | 161 (90.96%) | 16 (9.04%) | 143 (77.3%) | 42 (22.7%) | 0.0007 |
| FAT3 | 160 (90.4%) | 17 (9.6%) | 147 (79.46%) | 38 (20.54%) | 0.00597 |
| HMCN1 | 154 (87.01%) | 23 (12.99%) | 154 (83.24%) | 31 (16.76%) | 0.39157 |
| OBSCN | 160 (90.4%) | 17 (9.6%) | 146 (78.92%) | 39 (21.08%) | 0.0041 |
| ZFHX4 | 160 (90.4%) | 17 (9.6%) | 148 (80%) | 37 (20%) | 0.0086 |
| RYR2 | 161 (90.96%) | 16 (9.04%) | 147 (79.46%) | 38 (20.54%) | 0.0035 |
| SPTA1 | 159 (89.83%) | 18 (10.17%) | 149 (80.54%) | 36 (19.46%) | 0.0197 |
| PIK3CA | 157 (88.7%) | 20 (11.3%) | 151 (81.62%) | 34 (18.38%) | 0.0815 |
| CSMD1 | 162 (91.53%) | 15 (8.47%) | 149 (80.54%) | 36 (19.46%) | 0.0043 |
| P value was obtained from the chi-square test between different riskscore subtypes. | | | | | |

**
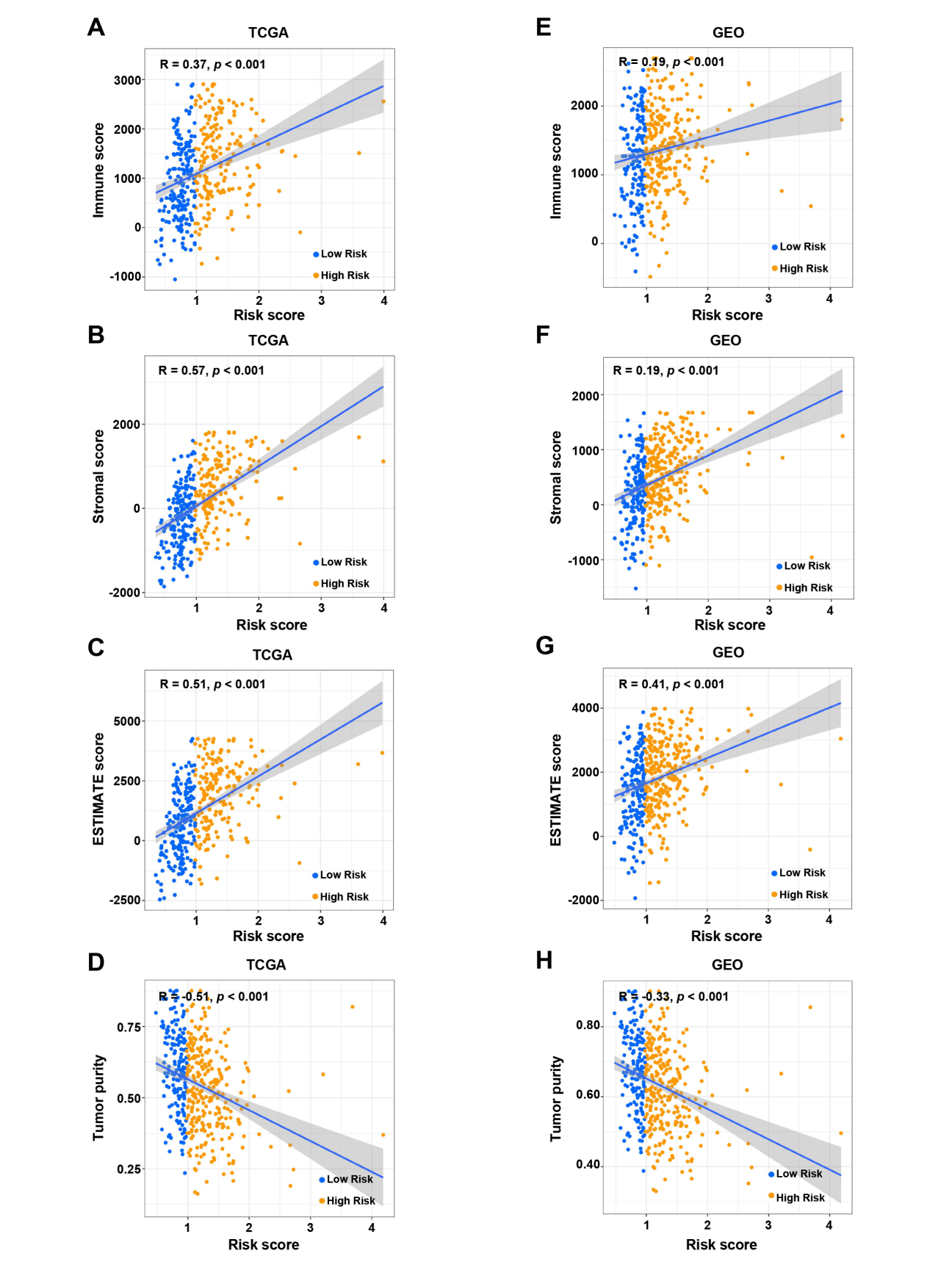
**

**Figure S1.** The correlation between risk score and immune score, stromal score, ESTIMATE score, and tumor purity in the screening and validation cohorts. (A) immune score, (B) stromal score, (C) ESTIMATE score, and (D) tumor purity in the screening cohort; (E) immune score, (F) stromal score, (G) ESTIMATE score, and (H) tumor purity in the validation cohort.

**
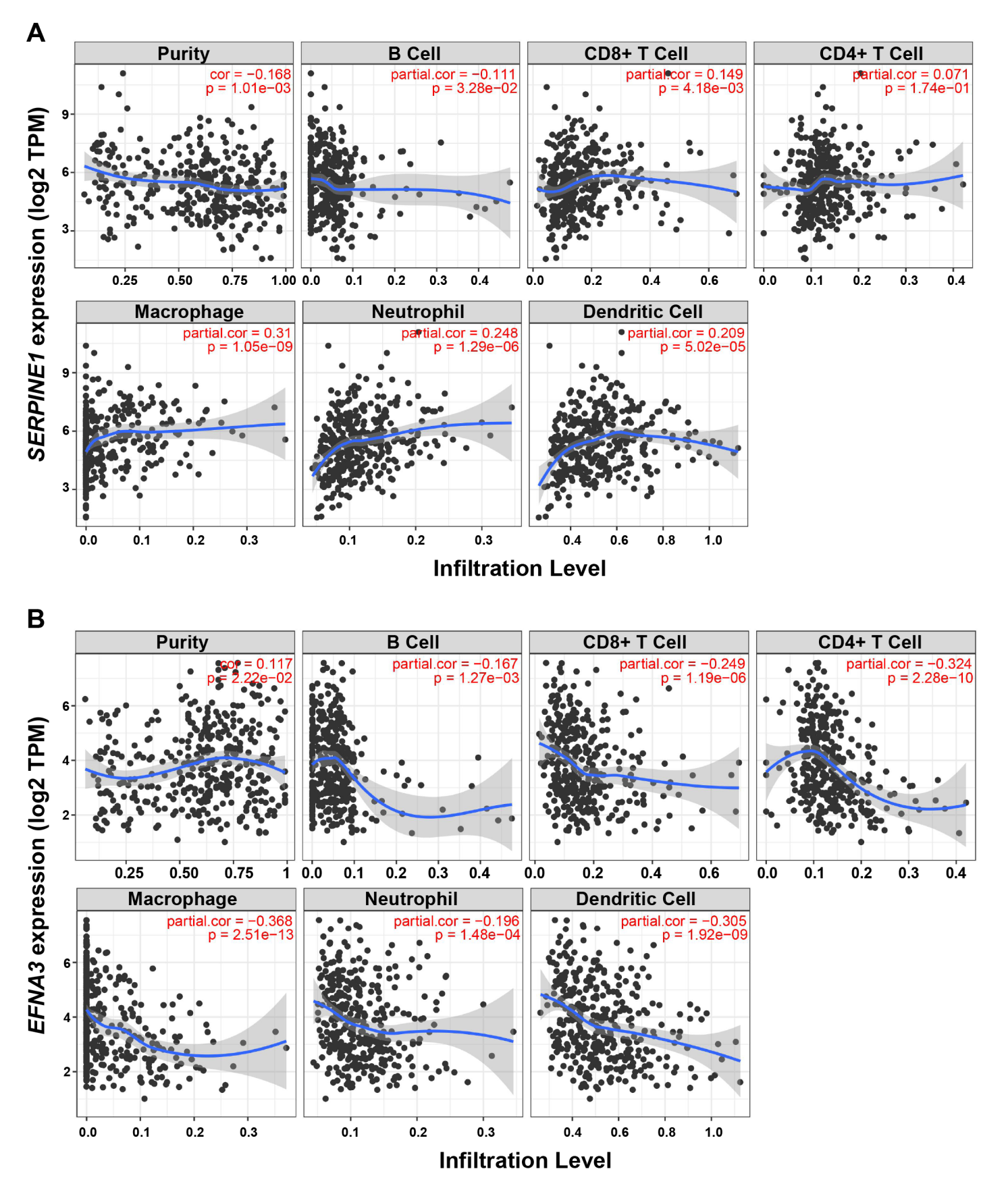
**

**Figure S2.** Correlation between gene expression and immune cell infiltration (TIMER). Correlation between the abundance of immune cells and the expression of (A) *SERPINE1*, and (B) *EFNA3*.

**
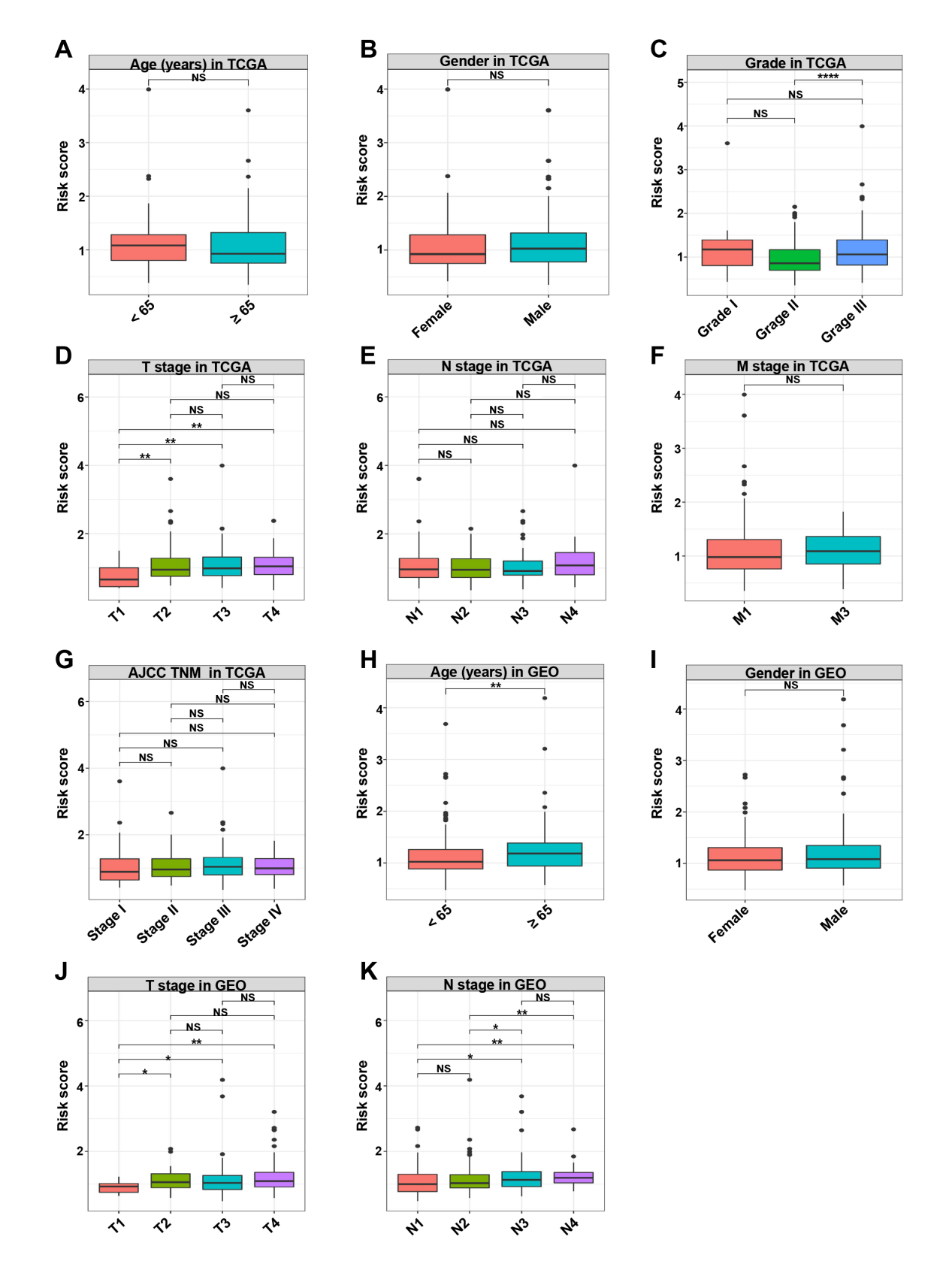
**

**Figure S3.** Relationship between the risk score and clinicopathological factors. (A) age, (B) gender, (C) grade, (D) T stage, (E) N stage, and (F) M stage, (G) AJCC TNM in the screening cohort; (H) age, (I) gender, (J) T stage, and (K) N stage in the validation cohort. (NS: *P* ≥ 0.05, **P* < 0.05, ***P* < 0.01, ****P* < 0.001)
